# Features of Thyroid Lymphoma: A Single-Center Experience

**DOI:** 10.3390/cancers18101574

**Published:** 2026-05-12

**Authors:** Enrico Battistella, Luca Pomba, Riccardo Toniato, Andrea Piotto, Ivana Cataldo, Mariella Lo Schirico, Antonio Toniato

**Affiliations:** 1 Endocrine Surgery Unit, Department of Surgery, Veneto Institute of Oncology, IOV-IRCCS, Via Gattamelata 64, 35128 Padova, Italy; 2 School of Medicine, University of Padua, Via Giustiniani 2, 35128 Padova, Italy; 3 Anatomy and Pathological Histology Unit, Veneto Institute of Oncology, IOV-IRCCS, Via Gattamelata 64, 35128 Padova, Italy; 4 Onco-Hematology, Department of Oncology, Veneto Institute of Oncology, IOV-IRCCS, Via Gattamelata 64, 35128 Padova, Italy; mariella.loschirico@iov.veneto.it

**Keywords:** thyroid lymphoma, thyroid cancer, thyroid surgery

## Abstract

Background: Primary thyroid lymphoma (PTL) is a rare malignancy, accounting for less than 5% of thyroid cancers and less than 2% of extranodal lymphomas. It predominantly affects older women and is strongly associated with autoimmune thyroiditis, particularly Hashimoto’s thyroiditis. Diagnosis is often challenging due to non-specific clinical, imaging, and cytological findings, and the role of surgery has progressively shifted from therapeutic to primarily diagnostic. Methods: We conducted a retrospective single-center case series including nine patients treated for PTL between 2015 and 2025 at a tertiary referral endocrine surgery center. An analysis was conducted on clinical presentation, pre-existing thyroid disease, diagnostic work-up, histopathological subtypes, treatment strategies and outcomes. All patients underwent preoperative ultrasound and fine-needle aspiration cytology (FNAC); surgical intervention was performed to confirm cytology results, when cytology was inconclusive or when compressive symptoms were present. Results: The cohort included six females and three males, with a median age of 65.2 years. Four patients had Hashimoto’s thyroiditis and three had multinodular goiter. FNAC was diagnostic or suggestive of lymphoma in three cases only, and surgical biopsy or thyroidectomy for a definitive diagnosis was performed in eight cases. One case started follow-up after cytology and flow cytometry. Histological subtypes were heterogeneous, including diffuse large B-cell lymphoma, Burkitt’s lymphoma, Hodgkin lymphoma, follicular lymphoma, high-grade B-cell lymphoma, and MALT lymphoma. Seven patients received combined chemoimmunotherapy. A complete response was obtained in eight patients, with a minimum follow-up of three years; one patient died of unrelated causes. Conclusions: PTL remains a rare and diagnostically challenging thyroid malignancy. FNAC alone is frequently insufficient, and surgical biopsy retains an important role in cases with high clinical suspicion or compressive symptoms. While surgery has limited therapeutic value, a multidisciplinary approach and timely, tailored treatment are crucial to achieving favorable outcomes.

## Simple Summary

Primary thyroid lymphoma (PTL) is a rare malignancy, comprising <5% of thyroid cancers and <2% of extranodal lymphomas, frequently associated with Hashimoto’s thyroiditis in elderly women. In this retrospective study of nine patients (2015–2025), diagnosis was often difficult due to non-specific findings. Fine-needle aspiration cytology (FNAC) was rarely diagnostic; thus, most patients required surgical biopsy or thyroidectomy for confirmation, particularly those with compressive symptoms. Histological subtypes varied between indolent and aggressive forms. Most patients received chemoimmunotherapy, resulting in excellent complete response rates. These outcomes confirm that surgery has a limited therapeutic role, while a multidisciplinary approach remains essential for timely diagnosis and effective management.

## 1. Introduction

Primary thyroid lymphoma (PTL) is an infrequent malignancy, accounting for roughly 1–5% of thyroid neoplasms and approximately 2% of extranodal lymphomas [1,2]. Clinically, PTL is characterized by lymphomatous involvement localized to the thyroid gland, occasionally extending to regional cervical lymph nodes [1,2,3]. The patient demographics typically shift toward older adults in their 60s and 70s, with a pronounced female predominance (ratio of ~4:1) [4,5].

The pathogenesis is inextricably linked to chronic inflammatory diseases; patients with Hashimoto’s thyroiditis face a risk nearly sixty times higher than the general population [6,7]. This association suggests that persistent immune stimulation triggers lymphoid hyperplasia, eventually facilitating malignant transformation within the thyroid microenvironment. While isolated cases have been documented in patients with Graves’ disease, there is currently no established causal link between PTL and conditions such as colloid goiter, ionizing radiation exposure, or specific chromosomal aberrations [6,8,9,10,11,12].

Treatment of PTL has evolved significantly. Surgical resection, once commonly performed for diagnostic and therapeutic purposes, now has a limited role, largely confined to airway stabilization or cases in which less invasive diagnostic methods are inconclusive [12,13,14,15,16].

The present study reports a case series from our institution, detailing the clinical characteristics, diagnostic work-up, histopathologic subtypes, therapeutic approaches and outcomes of patients with PTL managed over a defined period. Our findings may help to inform clinicians’ diagnostic vigilance and to guide selection of appropriate tailored treatment planning for this rare but potentially aggressive thyroid malignancy. This paper focuses exclusively on primary thyroid lymphoma.

## 2. Materials and Methods

In this retrospective single-center case series, clinical and demographic data from nine patients who underwent fine needle aspiration cytology or surgery (excisional biopsy or thyroidectomy) for primary thyroid lymphoma between 2015 and 2025 were extracted from a computerized endocrine-surgery registry. All procedures were performed at the Veneto Institute of Oncology, a tertiary referral center specialized in endocrine surgery. Each patient had been referred to the surgical unit for an evaluation of a rapidly enlarging cervical mass associated with compressive symptoms, including dysphagia, dysphonia, or dyspnea, or the performance of a fine needle aspiration cytology (FNAC) or surgical biopsy.

The variables collected included sex, age, the presence of pre-existing thyroid disorders (Hashimoto’s thyroiditis, Graves’ disease or multinodular goiter) and a significant family history of thyroid disease. All patients underwent a standardized preoperative evaluation comprising thyroid function tests (TSH, fT4 and fT3) and high-resolution cervical ultrasonography. Additional diagnostic investigations, including further imaging studies (e.g., computed tomography or magnetic resonance imaging) or supplementary laboratory analyses, were performed as clinically indicated on the basis of individual patient characteristics and preoperative findings.

The therapeutic approach adopted for each patient was taken into consideration and reported in our series, and patient outcomes, including survival, were assessed and incorporated in the analysis.

Written informed consent was obtained from every participant following a comprehensive explanation of the study’s purpose, procedures, and data-handling methods.

## 3. Results

The cohort comprised nine patients (six females and three males) with primary thyroid lymphoma (PTL) in a total of 830 cases of thyroid cancer (0.92%) managed between 2015 and 2025. The median age was 65.2 years with an age distribution ranging from 47 to 83 years.

**Figure 1:**
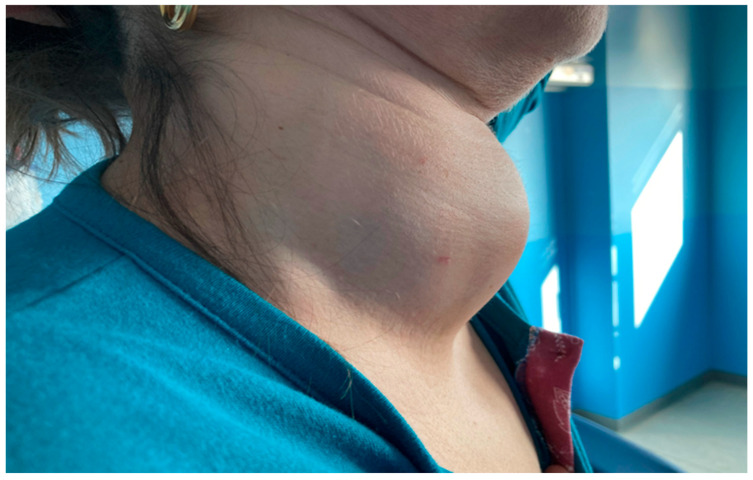
Enlarging neck mass in a patient with thyroid lymphoma.

All patients included in the study were euthyroid at the time of evaluation; four patients were receiving levothyroxine to restore normal thyroid hormone levels. With regard to their underlying thyroid conditions, one patient was diagnosed with multinodular goiter alone and one with Hashimoto’s thyroiditis (with a 4-year history), four patients had Hashimoto’s thyroiditis in a multinodular goiter (one with a 7-year history and three with a 3-year history) and two patients had a uninodular goiter (Figure 1).

Their thyroid functional status was assessed through standard biochemical testing as part of the preoperative workup.

**Figure 2:**
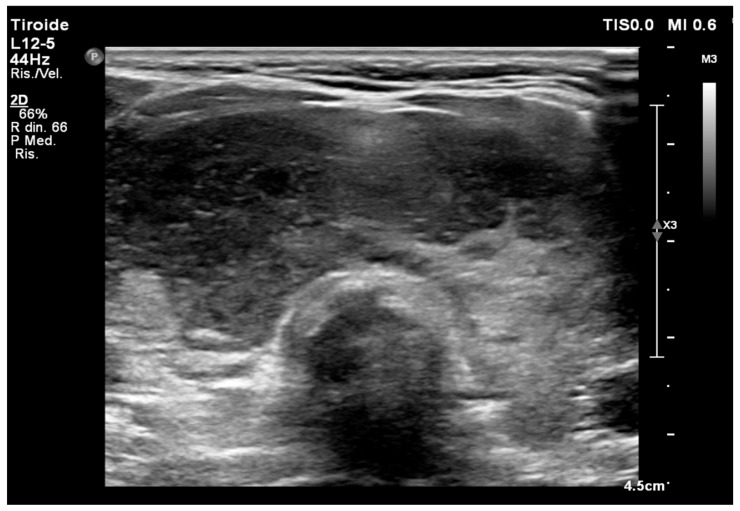
Ultrasound image of one patient of the study series with thyroid lymphoma. It appears as a markedly hypoechoic lesion, with ill-defined margins and absence of calcifications.

Preoperative ultrasonography was performed in all cases and patients presented a solid lesion having markedly hypoechoic echogenicity with increased intralesional vascularity compared to normal thyroid parenchyma, absence of intranodular calcifications, and variable margin definition (Figure 2).

Fine-needle aspiration cytology was performed on all patients, and the cytological report was suspicious for lymphoma in three cases. Three samples were non-diagnostic (Bethesda I), anaplastic thyroid cancer was suspected in one specimen, and two patients were diagnosed with a benign nodule (Bethesda II).

**Figure 3:**
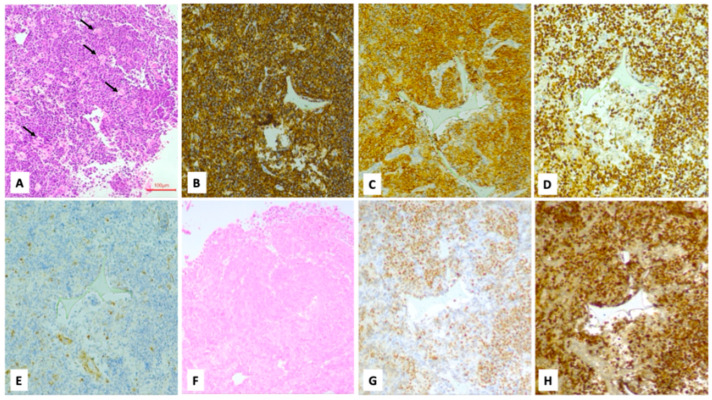
Burkitt’s lymphoma; thyroid biopsy. H&E (**A**): proliferation of intermediate-sized lymphoid elements with a round nucleus and some apoptosis, intermingled with scattered histiocytes with tingible bodies (indicated by arrows) with a starry sky appearance. Neoplastic cells stained positive for CD20 (**B**), CD10 (**C**), bcl6 (**D**), while they resulted negative for bcl2 (**E**) and EBER (**F**). C-MYC was detected in 80% of neoplastic cells (**G**), while the Ki-67 proliferation index (using clone MIB1) was 95% (**H**) (100× magnification).

**Figure 4:**
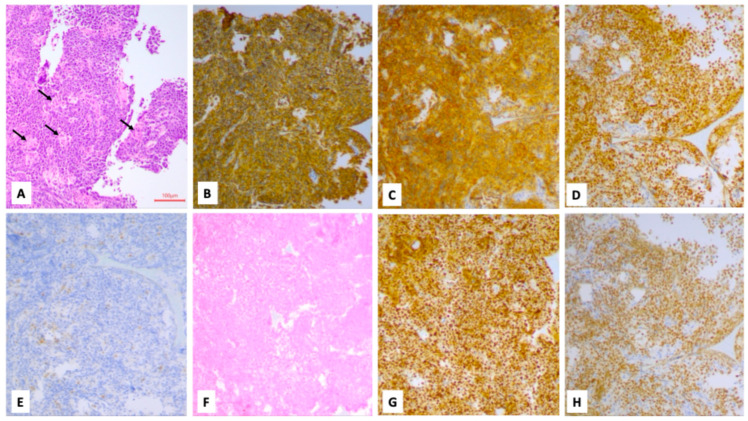
Burkitt’s lymphoma; thyroid biopsy. H&E (**A**): proliferation of intermediate sized lymphoid elements with a round nucleus and some apoptosis, intermingled with scattered histiocytes with tingible bodies (indicated by arrows) having a starry sky appearance. Neoplastic cells stained positive for CD20 (**B**), CD10 (**C**), bcl6 (**D**), while they resulted negative for bcl2 (**E**) and EBER (**F**). C-MYC was detected in more than 90% of neoplastic cells (**G**), while the Ki-67 proliferation index (using clone MIB1) was 95% (**H**) (100× magnification).

**Figure 5:**
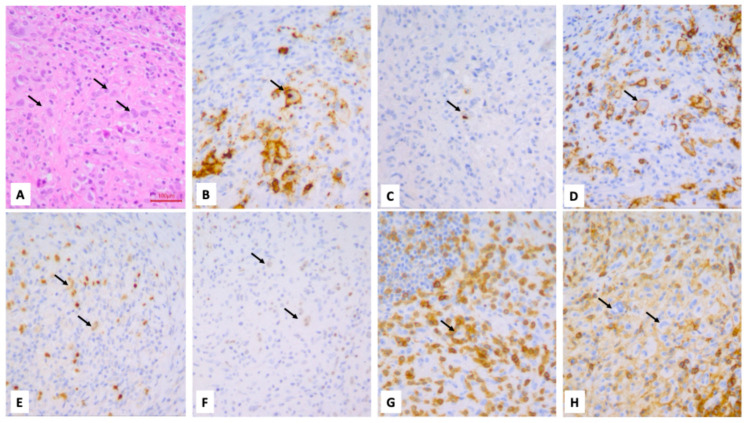
Classical Hodgkin lymphoma, nodular sclerosis variant; thyroid, nodulectomy. H&E (**A**): Thyroid parenchyma extensively infiltrated by a nodular proliferation of polymorphous lymphoid elements, with scattered large atypical Reed–Sternberg cells (arrows) admixed with small B and T-lymphocytes, rare plasma cells and several histiocytes. Neoplastic cells (arrows) stained positive for CD30 (**B**), CD15 (**C**), CD20 (focally) (**D**), Pax5 (**E**) and OCT2 (faint and focal positivity) (**F**), while they resulted negative for CD3 (positive T-lymphocytes around neoplastic cells) (**G**), CD45 (**H**) and EBER (200× magnification).

Surgery was performed on eight patients: five patients underwent an incisional biopsy, while the three patients with a cytological report of Bethesda I or II underwent a total thyroidectomy. The histological evaluation showed that two cases had diffuse large B-cell lymphoma (DLBCL), two cases were diagnosed with Burkitt’s lymphoma, one case had high-grade B-cell lymphoma (HGBCL) not otherwise specified (NOS), one case had follicular non-Hodgkin lymphoma, two cases had nodular sclerosis Hodgkin lymphoma and one case had MALT-lymphoma (Figure 3, Figure 4 and Figure 5).

All patients enrolled in the study underwent FDG-PET imaging as part of their diagnostic workup and staging assessment. This modality allowed for the evaluation of metabolically active disease sites and the detection of potential distant involvement.

In patients presenting with advanced-stage disease, FDG-PET findings revealed faint radiotracer uptake at the level of the left adrenal gland in two cases, suggestive of low-grade metabolic involvement. In one additional case, increased FDG uptake was observed in the bilateral laterocervical lymph nodes, consistent with nodal disease extension with a massive uptake of the thyroid gland (SUV 32.25).

One patient was diagnosed with low-grade follicular lymphoma by cytology and flow cytometry and started follow-up because it is considered an indolent, slow-growing disease by the current guidelines [17]. Indeed, four years later, the presence of periureteral disease was found during an emergency CT scan for anuria. The patient started six cycles of treatment with R–bendamustine (Rituximab–Bendamustine).

Eight patients received chemotherapy and immunotherapy. The patient with MALT lymphoma underwent a total thyroidectomy upfront because it appeared to be a multinodular goiter and then started follow-up.

The chemotherapy included six cycles of R-CHOP (cyclophosphamide, adriamycin, vincristine and prednisolone) with rituximab for patients with DLBCL, while the two patients diagnosed with Burkitt’s lymphoma received six cycles of R-Hyper CVAD-MA (rituximab, cyclophosphamide, vincristine, adriamycin, dexamethasone, and methotrexate). One patient with HGBCL-NOS was treated with six cycles of dose-adjusted EPOCH-R (etoposide, prednisone, vincristine, cyclophosphamide, doxorubicin, and rituximab) and high-dose methotrexate, and the two cases with nodular sclerosis Hodgkin lymphoma received six cycles of Brentuximab–AVD (brentuximab vedotin with doxorubicin, vinblastine, and dacarbazine).

Eight of the patients treated showed a complete therapeutic response. One patient with Burkitt’s lymphoma presented persistent FDG uptake only in the left thyroid lobe. For this reason, she underwent a thyroid lobectomy, and the histological examination only found fibrotic tissue.

Eight patients are still alive with a mean follow-up of 44 months (minimum 6 months, maximum 84 months) without any signs of persistence or recurrence of disease. One patient died of stroke (Table 1).

## 4. Discussion

Thyroid cancer is the most common endocrine malignancy, and primary thyroid lymphoma (PTL) accounts for less than 5% of all thyroid malignancies and approximately 3% of all non-Hodgkin lymphomas [1,2,3]. Between 2015 and 2025, we managed a total of 830 cases of thyroid cancer, of which only nine were identified as primary thyroid lymphoma (PTL), corresponding to an incidence of 0.92%. This finding is fully consistent with the rates reported in the literature and confirms the rarity of this type of malignancy [1,2,3].

As a general principle, the thyroid gland normally lacks significant lymphoid tissue. However, when pathological processes arise, lymphocytes can begin to infiltrate the gland, creating an environment that facilitates disease progression [11]. One of the best-known risk factors in this setting is autoimmune chronic lymphocytic thyroiditis, commonly known as Hashimoto’s thyroiditis. Individuals with this condition exhibit a markedly high risk of developing primary thyroid lymphoma (PTL) estimated to be 40 to 80 times higher than that of the general population [6,12,13].

Several studies report that Hashimoto’s thyroiditis is present in more than 90% of PTL cases, underscoring the strength of this association. The probable explanation for this close link lies in the persistent antigenic stimulation that characterizes chronic autoimmune inflammation, which may drive the lymphoid cells toward malignant transformation over time [6,18,19,20]. In our series, five patients have HT, representing 55.5% of the sample and confirming HT as a risk factor for primary thyroid lymphoma.

The most common histotype of primary thyroid lymphoma belongs to the category of B-cell non-Hodgkin lymphomas (98%): the most prevalent subtype is diffuse large B-cell lymphoma (DLBCL, 60–80% of cases), followed by mucosa-associated lymphoid tissue (MALT, 10–23% of cases). Other histological types include follicular lymphoma (10% of cases), followed by small lymphocytic lymphoma and chronic lymphocytic lymphoma (~3% of cases), Hodgkin lymphoma (2% of cases), T-cell lymphomas, and lymphoblastic lymphomas. The aggressiveness of the clinical presentation depends on its histology, with DLBCL displaying a more aggressive behavior than indolent lymphomas like MALT lymphoma or follicular lymphomas. In our study, we did not find a significant prevalence of any particular type of PTL [8,21,22].

The duration of symptoms before diagnosis can range widely from a few days to several years. Clinically, PTL often presents dramatically, with many patients experiencing a rapidly enlarging neck mass over weeks to months. This presentation may be accompanied by obstructive symptoms such as dyspnea, stridor, dysphagia, or hoarseness due to compression of the trachea or recurrent laryngeal nerve. Systemic B symptoms (fever, night sweats, weight loss, fatigue or pruritus) are less common but may occur in aggressive subtypes. Since these symptoms overlap with those of more prevalent thyroid diseases including anaplastic thyroid carcinoma, or benign goiters, initial clinical suspicion may be low, potentially delaying diagnosis [20].

There are no definitive pathognomonic markers in thyroid lymphomas; consequently, thyroid ultrasound is the recommended initial imaging method, although it frequently mimics other inflammatory or neoplastic conditions [23,24].

Fine needle aspiration cytology (FNAC) remains the primary diagnostic tool for the initial evaluation of thyroid lesions. However, the lack of pathognomonic features in PTL often complicates the cytological assessment, presenting significant hurdles for achieving a definitive diagnosis. While no large-scale randomized trials evaluating FNAC accuracy specifically for PTL have been conducted, data from several small-scale retrospective series indicate that diagnostic performance has improved. The integration of ancillary techniques—including flow cytometry, immunocytochemistry, and polymerase chain reaction (PCR)—has notably enhanced both the sensitivity and the specificity of the procedure [23,24,25,26].

We failed to obtain a satisfactory cytological report in six cases (66.6%), making it necessary to proceed with a surgical biopsy or a thyroidectomy [19]. In our opinion, it is mandatory to proceed with a surgical approach to avoid misdiagnosis and a diagnostic delay that could negatively affect the patient’s overall prognosis and reduce the possibility of starting an appropriate, targeted treatment in time. Such delays may allow the disease to progress unchecked, ultimately limiting therapeutic options and potentially worsening clinical outcomes. The therapeutic value of surgery in primary thyroid lymphoma remains controversial, as evidence from large referral centers does not support a benefit in terms of survival. Surgical management has not been shown to improve overall survival. The largest available series, reported by the Mayo Clinic and including 62 patients with PTL, demonstrated that total thyroidectomy followed by adjuvant radiotherapy did not ensure a higher survival rate than diagnostic biopsy combined with radiotherapy in patients with stage IE or IIE disease [27,28]. On this basis, we discouraged routine thyroidectomy, highlighting both the lack of benefit for survival and the potential morbidity associated with surgical procedures. Nevertheless, surgical intervention may still play a limited role in selected cases, such as patients with MALT lymphoma.

Following confirmation of the diagnosis, a multidisciplinary management strategy should be immediately adopted. A coordinated effort involving medical oncologists, radiation oncologists, and endocrinologists ensures that staging workup and therapeutic interventions proceed without delay [4].

In this case series, three patients underwent total thyroidectomy as there was no definitive preoperative diagnosis due to an unconclusive FNAC or benign cytological report and they presented compressive symptoms that interfered decisively with their quality of life. One patient with a benign cytology was supposed to undergo total thyroidectomy for multinodular goiter but, due to adhesions between the thyroid gland and the prethyroid muscles, and the surrounding anatomical structures, it was only possible to perform a surgical biopsy. Another patient was subjected to a thyroid lobectomy after chemotherapy for the persistence of the disease documented by a FDG PET-CT scan after a multidisciplinary discussion with the hematologist, surgeon, oncologist and radiologist. The histological examination only found fibrotic tissue.

FDG-PET plays a central role in the diagnosis, staging, response assessment, prognostication, and surveillance of high-grade lymphomas, significantly influencing clinical management [29]. Although imaging techniques such as ultrasound, CT, and [18F]FDG-PET/CT are fundamental for the initial detection and staging assessment of PTL, definitive diagnosis ultimately relies on histopathological evaluation obtained through surgical or core-needle biopsy, coupled with immunohistochemical analysis, underscoring the pivotal role of tissue sampling over imaging alone.

The small sample size represents a major limitation and reflects the rarity of PTL [30,31]. Additionally, the heterogeneity of histological subtypes and treatment strategies within such a small cohort further limits the ability to draw subtype-specific conclusions.

Larger, prospective, multi-center studies are therefore needed to validate these findings and to better define optimal diagnostic and therapeutic strategies for primary thyroid lymphoma.

## 5. Conclusions

Primary thyroid lymphoma is a rare and often underrecognized thyroid malignancy whose diagnosis may be challenging because of its non-specific clinical, laboratory, and imaging features. Our single-center experience confirms the low incidence of PTL among thyroid cancers and highlights its prevalence in older patients, frequent association with autoimmune thyroid disease, and heterogeneous histopathological spectrum. In our series, fine-needle aspiration cytology alone was insufficient to establish a definitive diagnosis in a substantial proportion of cases, reinforcing the limitations of FNAC in this setting and the potential need for surgical biopsy when clinical suspicion is high. Although surgery no longer plays a primary therapeutic role in PTL, our findings support its selective use as a diagnostic and, in carefully chosen cases, symptom-relieving strategy—particularly in patients with rapidly progressing compressive symptoms or inconclusive cytology. Ultimately, the management of primary thyroid lymphoma requires a multidisciplinary approach involving endocrinologists, surgeons, hematologists, oncologists, radiologists, and pathologists. Early recognition, accurate histological classification, and tailored treatment planning are essential to optimize clinical outcomes.

## Figures and Tables

**Table 1:** Clinical features of patients enrolled in this study.

Case ID	Sex	Age (Years)	Thyroid Disease	FNAC	Thyroid Surgery	Final Diagnosis	Stage	Treatment	Status
1	M	57	Multinodular MNG	Bethesda 1	Total thyroidectomy	HGBCL	IE	Da-Epoch-R + HD-MTX	Alive with no evidence of disease after 6 months
2	F	70	HT	Highly suspicious Lymphoproliferative disease	/	follicular NHL	IIIa	R–Bendamustine	Alive with no evidence of disease after 4 years
3	F	47	HT in MNG	Highly suspicious Lymphoproliferative disease	Surgical biopsy	Burkitt’s lymphoma	IVa	R-Hyper-CVAD/HD-MA	Alive with no evidence of disease after 3 years
4	F	74	UG	Highly suspicious Lymphoproliferative Disease	Surgical biopsy	Burkitt’s lymphoma	IVa	R-Hyper-CVAD/HD-MA	Alive with no evidence of disease after 3 years
5	M	83	UG	Anaplastic Thyroid cancer	Surgical biopsy	DLBCL	IIa	R-CHOP	Alive with no evidence of disease after 2 years
6	F	56	UG	Bethesda 1 Poorly differentiated epithelial neoplasm	Surgical biopsy	nodular sclerosis HL	IVb	Brentuximab–AVD	Alive with no evidence of disease after 3 years
7	F	50	MNG	Bethesda 2	Surgical biopsy	nodular sclerosis HL	IIa	Brentuximab–AVD	Alive with no evidence of disease after 7 years
8	M	70	HT in MNG	Bethesda 2	Total thyroidectomy	MALT lymphoma	/	/	Alive with no evidence of disease after 7 years
9	F	80	HT	Bethesda 1	Total thyroidectomy	DLBCL	IIa	R-CHOP	Died of stroke

HGBCL = high-grade B-cell lymphoma, NHL = non-Hodgkin lymphoma, DLBCL = diffuse large B-cell lymphoma, HT = Hashimoto’s thyroiditis, MNG = multinodular goiter, UG = uninodular goiter.

## Data Availability

Data can be found in the patients’ medical records.
